# Supplementary material for: Psychological interventions for weight reduction and sustained weight reduction in adults with overweight and obesity: a scoping review protocol
Source: BMJ Open. 2023 Dec 7;13(12):e075364. doi: 10.1136/bmjopen-2023-075364 (PMC10711885; doi:10.1136/bmjopen-2023-075364)
Supplement: Supplementary data [file bmjopen-2023-075364supp001.pdf]

Supplementary material 1
APPENDIX A
Example search strategy

Ovid MEDLINE(R) ALL <1946 to February 27, 2023>

1 Obesity/ 211963
2 Adiposity/ 15199
3 Weight Gain/ 35111
4 Weight Loss/ 42881
5 body mass index/ 147269
6 (obes* or adipos* or overweight* or over weight*).tw. 466238
7 (weight adj (reduc* or loss or losing or lose or maint* or decreas* or watch* or diet* or control* or manage*)).tw. 127938
8 or/1-7 672434
9 exp Psychotherapy/ 216317
10 (psychological therap* or psychological service* or psychological intervention* or psychological treatment* or psychotherap*).tw. 61353
11 counse?ling.tw. 112154
12 (interpersonal adj therap*).tw. 434
13 aversion therap*.tw. 187
14 behavio?r therap*.tw. 8063
15 behavio?r modific*.tw. 3108
16 colo?r therap*.tw. 27
17 (cognitiv* adj1 therap*).tw. 4261
18 gestalt therap*.tw. 94
19 music therap*.tw. 3010
20 milieu therap*.tw. 314
21 (assert* adj training).tw. 324
22 (nondirectiv* therap* or non directiv* therap*).tw. 47
23 ((problem solving or problemsolving) adj therap*).tw. 504
24 ((self control or selfcontrol) adj therap*).tw. 22
25 person cent*.tw. 8676
26 client cent*.tw. 1829
27 paradoxic* techni*.tw. 7
28 rational emoti*.tw. 289
29 reality therap*.tw. 189
30 (relax* adj train*).tw. 1436
31 mindfulness.tw. 11581
32 (sociotherap* or socio therap*).tw. 288
33 (socioenvironment* or socio environment*).tw. 1681
34 supportiv* therap*.tw. 5428
35 humanistic therap*.tw. 26
36 (person centered therap* or person centred therap*).tw. 42
37 psychodynamic therap*.tw. 619
38 motivational interviewing.tw. 4826
39 hypnotherapy.tw. 1216
40 or/9-39 376330
41 (Randomized Controlled Trial or Controlled Clinical Trial or Pragmatic Clinical Trial or Clinical Study or Adaptive Clinical Trial or Equivalence Trial).pt. 684949

42 (Clinical Trial or Clinical Trial, Phase I or Clinical Trial, Phase II or Clinical Trial, Phase III or Clinical Trial, Phase IV or Clinical Trial Protocol).pt. 608099
43 Multicenter Study.pt. 331074
44 Clinical Studies as Topic/ 782
45 exp Clinical Trial/ or exp Clinical Trials as Topic/ or Clinical Trial Protocol/ or Clinical Trial Protocols as Topic/ or exp "Clinical Trial (topic)"/ 1265276
46 Multicenter Study/ or Multicenter Studies as Topic/ or "Multicenter Study (topic)"/ 350118
47 Randomization/ 106905
48 Random Allocation/ 106905
49 Double-Blind Method/ 174454
50 Double Blind Procedure/ 0
51 Double-Blind Studies/174454
52 Single-Blind Method/ 32528
53 Single Blind Procedure/ 0
54 Single-Blind Studies/ 32528
55 Placebos/ 35925
56 Placebo/ 0
57 Control Groups/ 1911
58 Control Group/ 1911
59 Cross-Over Studies/ or Crossover Procedure/ 54748
60 (random* or sham or placebo*).ti,ab,hw,kf. 1779092
61 ((singl* or doubl*) adj (blind* or dumm* or mask*)).ti,ab,hw,kf. 264573
62 ((tripl* or trebl*) adj (blind* or dumm* or mask*)).ti,ab,hw,kf. 1544
63 (control* adj3 (study or studies or trial* or group*)).ti,ab,hw,kf. 1894433
64 (clinical adj3 (study or studies or trial*)).ti,ab,hw,kf. 1411390
65 (Nonrandom* or non random* or non-random* or quasi-random* or quasirandom*).ti,ab,hw,kf. 53387
66 (phase adj3 (study or studies or trial*)).ti,ab,hw,kf. 174884
67 ((crossover or cross-over) adj3 (study or studies or trial*)).ti,ab,hw,kf. 75902
68 ((multicent* or multi-cent*) adj3 (study or studies or trial*)).ti,ab,hw,kf. 401960
69 allocated.ti,ab,hw. 81914
70 ((open label or open-label) adj5 (study or studies or trial*)).ti,ab,hw,kf. 43646
71 ((equivalence or superiority or non-inferiority or noninferiority) adj3 (study or studies or trial*)).ti,ab,hw,kf. 11683
72 (pragmatic study or pragmatic studies).ti,ab,hw,kf. 575
73 ((pragmatic or practical) adj3 trial*).ti,ab,hw,kf. 7471
74 ((quasiexperimental or quasi-experimental) adj3 (study or studies or trial*)).ti,ab,hw,kf. 11671
75 trial.ti,kf. 300216
76 or/41-75 3732550
77 8 and 40 and 76 5462
78 limit 77 to English language 5302
