# Supplementary material for: Psychological interventions for weight reduction and sustained weight reduction in adults with overweight and obesity: a scoping review protocol
Source: BMJ Open. 2023 Dec 7;13(12):e075364. doi: 10.1136/bmjopen-2023-075364 (PMC10711885; doi:10.1136/bmjopen-2023-075364)
Supplement: Author's manuscript [file bmjopen-2023-075364.draft_revisions.pdf]

BMJ Open is committed to open peer review. As part of this commitment we make the peer review history of every article we publish publicly available.

When an article is published we post the peer reviewers' comments and the authors' responses online. We also post the versions of the paper that were used during peer review. These are the versions that the peer review comments apply to.

The versions of the paper that follow are the versions that were submitted during the peer review process. They are not the versions of record or the final published versions. They should not be cited or distributed as the published version of this manuscript.

BMJ Open is an open access journal and the full, final, typeset and author-corrected version of record of the manuscript is available on our site with no access controls, subscription charges or pay-per-view fees (http://bmjopen.bmj.com).

If you have any questions on BMJ Open's open peer review process please email info.bmjopen@bmj.com

BMJ Open

Psychological interventions for weight reduction and sustained weight reduction in adults with overweight and obesity: a scoping review protocol.

Journal:	*BMJ Open*
Manuscript ID	bmjopen-2023-075364
Article Type:	Protocol
Date Submitted by the Author:	05-May-2023

Complete List of Authors:	Hamer, Oliver; University of Central Lancashire<br>Bray, Emma; University of Central Lancashire, Primary Care Clinical Sciences<br>Harris, Cath; University of Central Lancashire,<br>Blundell, Amy; University of Central Lancashire<br>Kuroski, Jennifer A; University of Central Lancashire<br>Schneider, Emma; Liverpool University Hospitals NHS Foundation Trust<br>Watkins, Caroline; University of Central Lancashire, Faculty of Health and Wellbeing<br>Clegg, Andrew; University of Central Lancashire

Keywords:	Obesity, PUBLIC HEALTH, Quality of Life, PSYCHIATRY, Systematic Review, Clinical Decision-Making

SCHOLARONE™
Manuscripts

Title page – BMJ Open

TITLE

Psychological interventions for weight reduction and sustained weight reduction in adults with overweight and obesity: a scoping review protocol.

AUTHORS

1. *Dr Oliver Hamer, Senior RA, University of Central Lancashire, Preston, Lancashire, PR1 2HE. United Kingdom.
2. Dr Emma Bray, Senior Research Fellow, University of Central Lancashire, Preston, Lancashire, PR1 2HE. United Kingdom.
3. Catherine Harris, Information Specialist, University of Central Lancashire, Preston, Lancashire, PR1 2HE. United Kingdom.
4. Amy Blundell, Public Advisor, University of Central Lancashire - Comensus patient and public advisor group, Preston, Lancashire, PR1 2HE. United Kingdom.
5. Jennifer Kuroski, Research Associate, University of Central Lancashire, Preston, Lancashire, PR1 2HE. United Kingdom.
6. Emma Schneider, Advanced weight Management Specialist, Liverpool University Hospitals NHS Foundation Trust, Prescot Street, Liverpool, Merseyside, L7 8XP
7. Professor Dame Caroline Watkins, Professor of Stroke and Older People's Care, University of Central Lancashire, Preston, Lancashire, PR1 2HE. United Kingdom.
8. Professor Andrew Clegg, Professor of Health Services Research, University of Central Lancashire, Preston, Lancashire, PR1 2HE. United Kingdom.

*Corresponding author, Oliver Hamer, OHamer@uclan.ac.uk

KEYWORDS

Obesity; Body weight; Weight loss; Psychological intervention; Scoping review.

ABSTRACT

Introduction: Overweight and obesity are growing public health problems worldwide. Both diet and physical activity have been the primary interventions for weight reduction over the past decade. With increasing rates of overweight and obesity, it is evident that a primary focus on diet and exercise has not been wholly effective. There is now a case to explore other weight management strategies, focusing on psychological factors that may underpin overweight and obesity. Psychological therapy interventions are gaining recognition for their effectiveness in addressing underlying emotional factors and promoting weight loss. However, there is a dearth of literature that has mapped the types of psychological interventions and the characteristics of these interventions as a means of achieving weight reduction and sustained weight reduction in adults with overweight or obesity.

Methods and analysis: The review will combine the methodology outlined by Arksey and O'Malley with the PRISMA-ScR guidelines. A total of six databases will be searched using a comprehensive search strategy. Intervention studies will be included if participants are 18 years and over, classified as overweight or obese (≥BMI 25kg/m^2), and have received a psychological therapy intervention. The review will exclude studies that are not available in

For peer review only

English, not full text, none peer reviewed or combine a lifestyle and/or pharmacological intervention with a psychological intervention. Data will be synthesised using a narrative synthesis approach.

Ethics and dissemination: Ethical approval is not required to conduct this scoping review. The findings will be disseminated through journal publication(s), social media, and a lay summary for key stakeholders.

ARTICLE SUMMARY

Strengths and limitations of this study

- A comprehensive literature search of six electronic databases has been designed collaboratively with a skilled information specialist.
- The protocol for the review has been co-developed with a public advisor who has over ten years' experience living with obesity.
- The scoping review will provide an up-to-date synthesis on psychological interventions for weight loss, and may help to identify gaps in the current evidence base.
- The scoping review will be limited to comparisons with other psychological interventions, usual care, or no intervention, and will not therefore include a comparison with other active lifestyle interventions (unless classified as usual care).

WORDCOUNT

2498

For peer review only

Manuscript – BMJ Open

INTRODUCTION

Obesity is a growing public health problem worldwide (1). It has recently been recognised (and classified) by The Obesity Society and the European Commission as a chronic disease (2-5). Globally, approximately 650 million adults and 340 million children and adolescents (ages 5-19) currently suffer from obesity (6). Obesity is known to increase the risks of a wide spectrum of comorbidities, including cardiovascular disease, type 2 diabetes, cancer, hypertension, and several types of musculoskeletal disorders (7, 8). It has a multifaceted aetiology, with factors such as genetics, medical conditions, stress and environmental factors contributing to the prevalence of obesity (1).

Energy balance (energy input from food consumption and energy expenditure from physical activity) is one of the key factors to weight maintenance and, hence, diet (energy intake) and physical activity levels (energy output) are key behaviour determinants of obesity (9). Over the last 20 years, diet and physical activity have been the primary interventions recommended for weight loss (10). However, despite the efficacy of these interventions, long-term weight loss maintenance remains a challenge, with many individuals regaining lost weight. (11) With global overweight and obesity rates continuing to rise, it is evident that dietary and physical activity interventions have not been wholly effective (12). The lack of longer term (or population) success associated with these interventions may be due to a primary focus on increasing energy expenditure and reducing dietary intake (13). While these factors are critical for weight loss, they ignore the complex psychological, emotional, and behavioural factors that underpin overweight and obesity (6, 14-16). These factors can contribute to disordered eating, low self-esteem, depression, anxiety, and stress (which may lead to weight gain) (14, 17). Furthermore, these interventions often ignore the environmental factors that contribute to weight gain, such as food accessibility, social support, and cultural norms (18). With the previous emphasis on diet and physical activity interventions seemingly having little impact on population prevalence of overweight and obesity, there is now a case to explore additional weight management strategies, focusing on the psychological factors that may underpin overweight and obesity (16, 19).

In recent years, there has been increasing recognition of the importance of psychological interventions for weight reduction in adults with overweight and obesity (20). Psychological interventions address the underlying psychological and behavioural factors that contribute to excessive weight (21). These interventions support individuals to develop skills and strategies to cope with negative emotions and cognitions, modify unhelpful behaviours, and promote healthy lifestyle changes (20, 21). They also aim to increase self-efficacy, self-regulation, and self-awareness, which are critical for long-term weight reduction and maintenance (22). By targeting the psychological and behavioural factors that contribute to overweight and obesity, these interventions may be more effective for long-term weight loss maintenance compared to diet and physical activity interventions alone (23). These interventions may be delivered individually or in groups, in clinical, community, workplace, and online settings, and include

For peer review only

a variety of components such as cognitive behaviour change, mindfulness, relaxation and motivation (21, 22).

As research in the field of weight reduction and management continues to evolve, it is important to identify novel interventions (24). This is particularly important for psychological interventions, which are often overlooked in favour of more traditional approaches such as diet and exercise (22). Several systematic reviews have been conducted to assess the effectiveness of psychological interventions for weight loss in overweight or obese adults (21, 22). However, these reviews are limited by population (including only adults with specific medical conditions), inclusion of interventions (including only one type of psychological therapy intervention), or being substantially outdated (21, 22, 25). With the rapid development of new technology (e.g., digital health technologies) and therapeutic techniques, coupled with the ever-increasing rates of overweight and obesity, a scoping review is now needed to map the different types of psychological interventions and their core characteristics, and identify evidence gaps and areas for future research in this field (26).

Aim

To explore psychological interventions for adults with overweight or obesity as a means of achieving sustained weight reduction.

Objectives

1. To generate a comprehensive map of the types of psychological interventions for weight reduction and sustained weight reduction in adults with overweight or obesity.
2. To identify the core intervention characteristics including theoretical foundations, techniques, setting, duration, mode, number of sessions and provider of psychological interventions for weight reduction and sustained weight reduction in adults with overweight or obesity.
3. To identify study characteristics such as length of follow up, and outcomes of studies which include psychological interventions for weight reduction and sustained weight reduction in adults with overweight or obesity.
4. To generate a comprehensive map of the types of usual care for weight reduction and sustained weight reduction in adults with overweight or obesity.
5. To identify the core characteristics including components, setting, duration, mode, and providers of usual care for weight reduction and sustained weight reduction in adults with overweight or obesity.
6. To identify evidence gaps and areas for future research in the field of psychological interventions for weight reduction and sustained weight reduction in adults with overweight and obesity.

METHODS AND ANALYSIS

Study design

For peer review only

The study will follow a scoping review design, following the framework set out by Arksey and O'Malley, which recommends a five-stage review process (27):

- Stage 1. Identifying the research question.
- Stage 2. Identifying relevant studies.
- Stage 3. Study selection.
- Stage 4. Charting the data.
- Stage 5. Collating, summarising, and reporting the results

We will not implement the optional sixth stage set out by Arksey and O'Malley as the first five stages are adequate to satisfy the reviews aims and objectives (19). The study will be reported in accordance with the Preferred Reporting Items for Systematic reviews and Meta-Analyses extension for Scoping Reviews (PRISMA-ScR) guidelines (28).

Search strategy

Six databases will be searched to identify relevant articles:

1. Cochrane Central Register of Controlled Trials (CENTRAL).
2. World Health Organization International Clinical Trials Registry Platform (ICTRP).
3. EMBASE.
4. MEDLINE.
5. CINAHL.
6. PsycInfo.

We will search the above databases and trials registries from their inception to the present. A search of clinical trials will be conducted through the CENTRAL and ICTRP databases to identify ongoing clinical trials. We will check the reference lists of all included studies and relevant review articles for additional studies.

The search strategy conducted in each database will be based on the following:

- Population: Adults with overweight or obesity (aged ≥18, with BMI ≥25kg/m^2).
- Intervention: Psychological interventions (e.g., cognitive behaviour therapy, mindfulness, motivational interviewing etc).
- Study type: Randomised controlled trials (RCTs) or non-randomised comparative intervention studies.

The search strategy, designed by the research team in collaboration with an expert information specialist, will be adapted for each database (See Appendix A for Ovid example). The search strategy was based on the strategy outlined in the Cochrane review by Shaw et al (2005), but with the addition of further search terms identified by the research team, and the CADTH search filter (to identify randomised and non-randomised controlled trials) (22, 29).

For peer review only

Study selection

All articles identified by the electronic database searches will be imported into Rayyan (web application). One reviewer will then independently screen the titles and abstracts of the search results for appropriate studies. Following initial screening, two reviewers will independently screen the full texts of all potentially eligible studies. The two reviewers will resolve disagreements through discussion with a third reviewer. Full-text articles that meet the inclusion criteria will be charted to summarise the findings. The study selection process will be recorded in sufficient detail using a PRISMA flow diagram, reporting reasons for exclusion.

Data extraction

One reviewer will independently extract data for the included studies using a piloted data extraction form. A second reviewer will verify the data extraction. Data will be extracted on the following: study methods, participants, interventions (e.g., type, setting, components), outcomes and any other information judged to be important to meet the objectives of the study.

Inclusion criteria

While a scoping review traditionally encompasses a wide range of literature, specific criteria will be employed to identify relevant articles for the aim and objectives of the study.

Types of studies

Randomised controlled trials (RCTs) and non-randomised comparative intervention studies will be included. We will only include studies reported in full text. Whereby we identify an abstract meeting the inclusion criteria, we will contact the study authors to establish if a full text is available.

Types of participants

Studies will be included if the participants are ≥18 years and were classified as overweight or obese (BMI ≥ 25kg/m^2). Within studies, BMI or the components of BMI, may be self-reported or measured. Studies will also be included when a sample includes participants with other classifications of BMI (e.g., underweight, healthy weight) if data relating to those with overweight or obesity can be extracted separately.

Studies will be included whereby participants with overweight or obesity have other co-morbidities (e.g., musculoskeletal pain, osteoarthritis, diabetes, hypertension, arthritis, hyperuricemia, gall bladder disease etc.), as BMI greater than 25kgm^2 has been associated with the incidence of multiple co-morbidities (30).

Types of interventions

Psychological therapy interventions will be defined as those interventions that involve meeting with a therapist (healthcare professional competent in providing psychological therapy) to discuss feelings and thoughts and how these affect behaviour and wellbeing (31). All psychological interventions will be considered for inclusion. Some of these interventions include cognitive behaviour therapy (CBT), mindfulness, psychodynamic therapies, motivational interviewing (MI), prolonged exposure therapy (PE), hypnotherapy, relaxation,

For peer review only

psychotherapy, rationale emotional therapy and systematic desensitisation. Individual and group therapies will be included.

We will only include studies of psychological therapies that are designed to reduce weight or increase health promoting behaviour for the purpose of sustained weight reduction (i.e., eating behaviour, or physical activity).

To identify psychological interventions for weight reduction and sustained weight reduction, we will include studies with the following comparisons:

- Psychological therapy versus no intervention.
- Psychological therapy versus usual care (as defined by study authors).
- Comparisons between different types of psychological therapy (e.g., CBT, mindfulness, motivational interviewing etc).

We will not compare psychological interventions with other active interventions (e.g., diet and exercise interventions), unless these are classified as usual care.

Outcomes

Information on the following study outcomes, where available, will be collected: body weight or indicator of body mass (e.g., body mass index), adverse events, quality of life, physical activity levels and eating behaviours.

Exclusion criteria

We will exclude studies that are not available in English, are not full text articles, and not peer reviewed articles (e.g., magazine articles, letters, editorials, newspaper, and commentary articles).

Data synthesis and analysis

A narrative synthesis will be employed to synthesise the data, prioritising and ordering data using the guidelines of the Synthesis WIthout Meta-analysis in systematic reviews (SWiM) (32).

Patient and public involvement (PPI)

A public advisor with more than ten years' experience of living with obesity has been involved in designing the review and this protocol paper, and will continue to have input throughout the project, including the determination of the objectives and improving the lay interpretability of the review. The public advisor will also help to co-develop the dissemination strategy.

ETHICS AND DISSEMINATION

For peer review only

Ethical approval is not required to conduct this scoping review. The findings will be submitted to a peer-reviewed journal for publication. A lay summary will also be prepared with the assistance of the PPI author and disseminated on social media and to other key stakeholders involved in the care of adults with weight concerns. Findings will also be shared on the National Institute for Health and Care Research, Applied Research Collaboration - Northwest Coast (NIHR ARC-NWC) social media pages.

FUNDING STATEMENT
This work was partly funded by the National Institute for Health and Care Research Applied Research Collaboration North West Coast (NIHR ARC NWC). The work was also partly funded by the University of Central Lancashire, Research Institute for Global Health and Wellbeing (LIFE). The views expressed are those of the authors and not necessarily those of the NHS, the NIHR, or the Department of Health and Social Care.

CONFLICT OF INTERESTS STATEMENT
The authors have no conflicts of interest to declare.

AUTHOR STATEMENT - CONTRIBUTIONS
OH was responsible for the conception, design and writing of the manuscript. CW, EB, and AC provided support throughout the conception and design. AC, EB and JK contributed to the writing of the manuscript. AB provided PPI input throughout the development of the protocol, including the outcomes and background. CH designed the search strategy and performed the preliminary database searches. ES provided clinical input to support the development of the protocol. All authors read and approved the final manuscript.

REFERENCES
1. Cruickshank R, Crummey A, Muir VH. Obesity: a growing problem. Br Dent J. 2021;230(11):687-8.
2. Jastreboff AM, Kotz CM, Kahan S, Kelly AS, Heymsfield SB. Obesity as a disease: the obesity society 2018 position statement. Obesity. 2019;27(1):7-9.
3. Allison DB, Downey M, Atkinson RL, Billington CJ, Bray GA, Eckel RH, et al. Obesity as a disease: a white paper on evidence and arguments commissioned by the Council of the Obesity Society. Obesity. 2008;16(6):1161.
4. Burki T. European Commission classifies obesity as a chronic disease. The Lancet Diabetes & Endocrinology. 2021;9(7):418.
5. Kushner R. Obesity 2021: Current Clinical Management of a Chronic, Serious Disease. J Fam Pract. 2021;70(6s):S35-s40.
6. Sørensen TIA, Martinez AR, Jørgensen TSH. Epidemiology of Obesity. Handb Exp Pharmacol. 2022;274:3-27.
7. Kyrou I, Christou A, Panagiotakos D, Stefanaki C, Skenderi K, Katsana K, et al. Effects of a hops (Humulus lupulus L.) dry extract supplement on self-reported depression, anxiety and stress levels in apparently healthy young adults: a randomized, placebo-controlled, double-blind, crossover pilot study. Hormones (Athens). 2017;16(2):171-80.
8. Dowd JB, Zajacova A. Long-term obesity and cardiovascular, inflammatory, and metabolic risk in U.S. adults. Am J Prev Med. 2014;46(6):578-84.

For peer review only

9. Hill JO, Wyatt HR, Peters JC. Energy balance and obesity. Circulation. 2012;126(1):126-32.
10. de Lannoy L, Cowan T, Fernandez A, Ross R. Physical activity, diet, and weight loss in patients recruited from primary care settings: An update on obesity management interventions. Obes Sci Pract. 2021;7(5):619-28.
11. Hall KD, Kahan S. Maintenance of Lost Weight and Long-Term Management of Obesity. Med Clin North Am. 2018;102(1):183-97.
12. Chooi YC, Ding C, Magkos F. The epidemiology of obesity. Metabolism. 2019;92:6-10.
13. Deslippe AL, Soanes A, Bouchaud CC, Beckenstein H, Slim M, Plourde H, et al. Barriers and facilitators to diet, physical activity and lifestyle behavior intervention adherence: a qualitative systematic review of the literature. International Journal of Behavioral Nutrition and Physical Activity. 2023;20(1):14.
14. Kumar R, Rizvi MR, Saraswat S. Obesity and Stress: A Contingent Paralysis. Int J Prev Med. 2022;13:95.
15. Puhl RM, Heuer CA. Obesity stigma: important considerations for public health. Am J Public Health. 2010;100(6):1019-28.
16. Hamer O, Larkin, D., Relph, N., & Dey, P. . Fear as a barrier to physical activity in young adults with obesity: a qualitative study. . Qualitative Research in Sport, Exercise and Health. 2021.
17. Torres SJ, Nowson CA. Relationship between stress, eating behavior, and obesity. Nutrition. 2007;23(11-12):887-94.
18. Hruby A, Hu FB. The Epidemiology of Obesity: A Big Picture. Pharmacoeconomics. 2015;33(7):673-89.
19. Hamer O, Larkin D, Relph N, Dey P. Fear-related barriers to physical activity among adults with overweight and obesity: A narrative synthesis scoping review. Obes Rev. 2021;22(11):e13307.
20. Barrett S, Begg S, O'Halloran P, Kingsley M. Integrated motivational interviewing and cognitive behaviour therapy for lifestyle mediators of overweight and obesity in community-dwelling adults: a systematic review and meta-analyses. BMC Public Health. 2018;18(1):1160.
21. Jacob A, Moullec G, Lavoie KL, Laurin C, Cowan T, Tisshaw C, et al. Impact of cognitive-behavioral interventions on weight loss and psychological outcomes: A meta-analysis. Health Psychol. 2018;37(5):417-32.
22. Shaw K, O'Rourke P, Del Mar C, Kenardy J. Psychological interventions for overweight or obesity. Cochrane Database Syst Rev. 2005(2):Cd003818.
23. Teixeira PJ, Carraça EV, Marques MM, Rutter H, Oppert J-M, De Bourdeaudhuij I, et al. Successful behavior change in obesity interventions in adults: a systematic review of self-regulation mediators. BMC Medicine. 2015;13(1):84.
24. Gómez Puente JM, Martínez-Marcos M. Overweight and obesity: effectiveness of interventions in adults. Enferm Clin (Engl Ed). 2018;28(1):65-74.
25. Palavras MA, Hay P, Filho CA, Claudino A. The Efficacy of Psychological Therapies in Reducing Weight and Binge Eating in People with Bulimia Nervosa and Binge Eating Disorder Who Are Overweight or Obese-A Critical Synthesis and Meta-Analyses. Nutrients. 2017;9(3).
26. Munn Z, Peters MDJ, Stern C, Tufanaru C, McArthur A, Aromataris E. Systematic review or scoping review? Guidance for authors when choosing between a systematic or scoping review approach. BMC Med Res Methodol. 2018;18(1):143.
27. Arksey H, O’Malley L. Scoping studies: towards a methodological framework. International Journal of Social Research Methodology. 2005;8:19 - 32.

28. Peters MDJ, Marnie C, Tricco AC, Pollock D, Munn Z, Alexander L, et al. Updated methodological guidance for the conduct of scoping reviews. JBI Evid Synth. 2020;18(10):2119-26.
29. CADTH. CADTH Search Filters Database CADTH Evidence Driven2023 [Available from: https://searchfilters.cadth.ca/link/34. .
30. Pi-Sunyer FX. Comorbidities of overweight and obesity: current evidence and research issues. Med Sci Sports Exerc. 1999;31(11 Suppl):S602-8.
31. NICE. Information for the public - Psychological therapy Online: National Institute for Health and Care Excellence; 2014 [Available from: https://www.nice.org.uk/guidance/cg178/ifp/chapter/psychological-therapy.
32. Campbell M, McKenzie JE, Sowden A, Katikireddi SV, Brennan SE, Ellis S, et al. Synthesis without meta-analysis (SWiM) in systematic reviews: reporting guideline. BMJ. 2020;368:l6890.

For peer review only

APPENDIX A

Example search strategy

<u>Ovid MEDLINE(R) ALL <1946 to February 27, 2023></u>

1 Obesity/ 211963
2 Adiposity/ 15199
3 Weight Gain/ 35111
4 Weight Loss/ 42881
5 body mass index/ 147269
6 (obes* or adipos* or overweight* or over weight*).tw. 466238
7 (weight adj (reduc* or loss or losing or lose or maint* or decreas* or watch* or diet* or control* or manage*)).tw. 127938
8 or/1-7 672434
9 exp Psychotherapy/ 216317
10 (psychological therap* or psychological service* or psychological intervention* or psychological treatment* or psychotherap*).tw. 61353
11 counse?ling.tw. 112154
12 (interpersonal adj therap*).tw. 434
13 aversion therap*.tw. 187
14 behavio?r therap*.tw. 8063
15 behavio?r modific*.tw. 3108
16 colo?r therap*.tw. 27
17 (cognitiv* adj1 therap*).tw. 4261
18 gestalt therap*.tw. 94
19 music therap*.tw. 3010
20 milieu therap*.tw. 314
21 (assert* adj training).tw. 324
22 (nondirectiv* therap* or non directiv* therap*).tw. 47
23 ((problem solving or problemsolving) adj therap*).tw. 504
24 ((self control or selfcontrol) adj therap*).tw. 22
25 person cent*.tw. 8676
26 client cent*.tw. 1829
27 paradoxic* techni*.tw. 7
28 rational emoti*.tw. 289
29 reality therap*.tw. 189
30 (relax* adj train*).tw. 1436
31 mindfulness.tw. 11581
32 (sociotherap* or socio therap*).tw. 288
33 (socioenvironment* or socio environment*).tw. 1681
34 supportiv* therap*.tw. 5428
35 humanistic therap*.tw. 26
36 (person centered therap* or person centred therap*).tw. 42
37 psychodynamic therap*.tw. 619
38 motivational interviewing.tw. 4826
39 hypnotherapy.tw. 1216
40 or/9-39376330
41 (Randomized Controlled Trial or Controlled Clinical Trial or Pragmatic Clinical Trial or Clinical Study or Adaptive Clinical Trial or Equivalence Trial).pt. 684949

For peer review only

42 (Clinical Trial or Clinical Trial, Phase I or Clinical Trial, Phase II or Clinical Trial, Phase III or Clinical Trial, Phase IV or Clinical Trial Protocol).pt. 608099
43 Multicenter Study.pt. 331074
44 Clinical Studies as Topic/ 782
45 exp Clinical Trial/ or exp Clinical Trials as Topic/ or Clinical Trial Protocol/ or Clinical Trial Protocols as Topic/ or exp "Clinical Trial (topic)"/ 1265276
46 Multicenter Study/ or Multicenter Studies as Topic/ or "Multicenter Study (topic)"/ 350118
47 Randomization/ 106905
48 Random Allocation/ 106905
49 Double-Blind Method/ 174454
50 Double Blind Procedure/ 0
51 Double-Blind Studies/174454
52 Single-Blind Method/ 32528
53 Single Blind Procedure/ 0
54 Single-Blind Studies/ 32528
55 Placebos/ 35925
56 Placebo/ 0
57 Control Groups/ 1911
58 Control Group/ 1911
59 Cross-Over Studies/ or Crossover Procedure/ 54748
60 (random* or sham or placebo*).ti,ab,hw,kf. 1779092
61 ((singl* or doubl*) adj (blind* or dumm* or mask*)).ti,ab,hw,kf. 264573
62 ((tripl* or trebl*) adj (blind* or dumm* or mask*)).ti,ab,hw,kf. 1544
63 (control* adj3 (study or studies or trial* or group*)).ti,ab,hw,kf. 1894433
64 (clinical adj3 (study or studies or trial*)).ti,ab,hw,kf. 1411390
65 (Nonrandom* or non random* or non-random* or quasi-random* or quasirandom*).ti,ab,hw,kf. 53387
66 (phase adj3 (study or studies or trial*)).ti,ab,hw,kf. 174884
67 ((crossover or cross-over) adj3 (study or studies or trial*)).ti,ab,hw,kf. 75902
68 ((multicent* or multi-cent*) adj3 (study or studies or trial*)).ti,ab,hw,kf. 401960
69 allocated.ti,ab,hw. 81914
70 ((open label or open-label) adj5 (study or studies or trial*)).ti,ab,hw,kf. 43646
71 ((equivalence or superiority or non-inferiority or noninferiority) adj3 (study or studies or trial*)).ti,ab,hw,kf. 11683
72 (pragmatic study or pragmatic studies).ti,ab,hw,kf. 575
73 ((pragmatic or practical) adj3 trial*).ti,ab,hw,kf. 7471
74 ((quasiexperimental or quasi-experimental) adj3 (study or studies or trial*)).ti,ab,hw,kf. 11671
75 trial.ti,kf. 300216
76 or/41-75 3732550
77 8 and 40 and 76 5462
78 limit 77 to English language 5302

For peer review only

BMJ Open

Psychological interventions for weight reduction and sustained weight reduction in adults with overweight and obesity: a scoping review protocol.

Journal:	*BMJ Open*
Manuscript ID	bmjopen-2023-075364.R1
Article Type:	Protocol
Date Submitted by the Author:	16-Sep-2023
Complete List of Authors:	Hamer, Oliver; University of Central Lancashire<br>Bray, Emma; University of Central Lancashire, Primary Care Clinical Sciences<br>Harris, Cath; University of Central Lancashire,<br>Blundell, Amy; University of Central Lancashire<br>Kuroski, Jennifer A; University of Central Lancashire<br>Schneider, Emma; Liverpool University Hospitals NHS Foundation Trust<br>Watkins, Caroline; University of Central Lancashire, Faculty of Health and Wellbeing<br>Clegg, Andrew; University of Central Lancashire
**Primary Subject Heading**:	Public health
Secondary Subject Heading:	Public health, Global health, Epidemiology
Keywords:	Obesity, PUBLIC HEALTH, Quality of Life, PSYCHIATRY, Systematic Review, Clinical Decision-Making

SCHOLARONE™
Manuscripts

Title page – BMJ Open

TITLE

Psychological interventions for weight reduction and sustained weight reduction in adults with overweight and obesity: a scoping review protocol.

AUTHORS

1. *Dr Oliver Hamer, Senior RA, University of Central Lancashire, Preston, Lancashire, PR1 2HE. United Kingdom.
2. Dr Emma Bray, Senior Research Fellow, University of Central Lancashire, Preston, Lancashire, PR1 2HE. United Kingdom.
3. Catherine Harris, Information Specialist, University of Central Lancashire, Preston, Lancashire, PR1 2HE. United Kingdom.
4. Amy Blundell, Public Advisor, University of Central Lancashire - Comensus patient and public advisor group, Preston, Lancashire, PR1 2HE. United Kingdom.
5. Jennifer Kuroski, Research Associate, University of Central Lancashire, Preston, Lancashire, PR1 2HE. United Kingdom.
6. Emma Schneider, Advanced weight Management Specialist, Liverpool University Hospitals NHS Foundation Trust, Prescot Street, Liverpool, Merseyside, L7 8XP
7. Professor Dame Caroline Watkins, Professor of Stroke and Older People's Care, University of Central Lancashire, Preston, Lancashire, PR1 2HE. United Kingdom.
8. Professor Andrew Clegg, Professor of Health Services Research, University of Central Lancashire, Preston, Lancashire, PR1 2HE. United Kingdom.

*Corresponding author, Oliver Hamer, OHamer@uclan.ac.uk

KEYWORDS

Obesity; Body weight; Weight loss; Psychological intervention; Scoping review.

ABSTRACT

Introduction: Overweight and obesity are growing public health problems worldwide. Both diet and physical activity have been the primary interventions for weight reduction over the past decade. With increasing rates of overweight and obesity, it is evident that a primary focus on diet and exercise has not resulted in sustained obesity reduction within the global population. There is now a case to explore other weight management strategies, focusing on psychological factors that may underpin overweight and obesity. Psychological therapy interventions are gaining recognition for their effectiveness in addressing underlying emotional factors and promoting weight loss. However, there is a dearth of literature that has mapped the types of psychological interventions and the characteristics of these interventions as a means of achieving weight reduction and sustained weight reduction in adults with overweight or obesity.

Methods and analysis: The review will combine the methodology outlined by Arksey and O'Malley with the PRISMA-ScR guidelines. A total of six databases will be searched using a comprehensive search strategy. Intervention studies will be included if participants are 18 years and over, classified as overweight or obese (≥BMI 25kg/m^2), and have received a

For peer review only - http://bmjopen.bmj.com/site/about/guidelines.xhtml

psychological therapy intervention. The review will exclude studies that are not available in English, not full text, none peer reviewed or combine a lifestyle and/or pharmacological intervention with a psychological intervention. Data will be synthesised using a narrative synthesis approach.

Ethics and dissemination: Ethical approval is not required to conduct this scoping review. The findings will be disseminated through journal publication(s), social media, and a lay summary for key stakeholders.

Strengths and limitations of this study

- A comprehensive literature search of six electronic databases has been designed collaboratively with a skilled information specialist.
- The protocol for the review has been co-developed with a public advisor who has over ten years' experience living with obesity.
- The scoping review will be limited to comparisons with other psychological interventions, usual care, or no intervention, and will not therefore include a comparison with other active lifestyle interventions (unless classified as usual care).
- Relevant papers written in other languages could be omitted from this review, given the inclusion criteria to only include papers written in English.

WORDCOUNT

2498

For peer review only - http://bmjopen.bmj.com/site/about/guidelines.xhtml

Manuscript – BMJ Open

INTRODUCTION

Obesity is a growing public health problem worldwide (1). It has recently been recognised (and classified) by The Obesity Society and the European Commission as a chronic disease (2-5). Globally, approximately 650 million adults and 340 million children and adolescents (ages 5-19) currently suffer from obesity (6). Obesity is known to increase the risks of a wide spectrum of comorbidities, including cardiovascular disease, type 2 diabetes, cancer, hypertension, and several types of musculoskeletal disorders (7-9). It has a multifaceted aetiology, with factors such as genetics, medical conditions, stress and environmental factors contributing to the prevalence of obesity (1).

For peer review only

Energy balance (energy input from food consumption and energy expenditure from physical activity) is one of the key factors to weight maintenance and, hence, diet (energy intake) and physical activity levels (energy output) are key behaviour determinants of obesity (10). Over the last 20 years, diet and physical activity have been the primary interventions recommended for weight loss (11). However, despite the efficacy of these interventions, long-term weight loss maintenance remains a challenge, with many individuals regaining lost weight. (12) With worldwide prevalence of overweight and obesity rates continuing to rise, it is evident that dietary and physical activity interventions have not resulted in a sustained obesity reduction within the global population (13). The lack of longer term (or population) success associated with these interventions may be due to a primary focus on increasing energy expenditure and reducing dietary intake (14). While these factors are critical for weight loss, they ignore the complex psychological (e.g., fears), emotional (e.g., stress), and behavioural factors (e.g., avoidance) that underpin overweight and obesity (6, 15-18). These factors can contribute to disordered eating, low self-esteem, depression, anxiety, and stress (which may lead to weight gain) (15, 19). Furthermore, these interventions often ignore the environmental factors that contribute to weight gain, such as food accessibility, social support, and cultural norms (20). With the previous emphasis on diet and physical activity interventions seemingly having little impact on population prevalence of overweight and obesity, there is now a case to explore additional weight management strategies, focusing on the psychological factors that may underpin overweight and obesity (17, 21).

In recent years, there has been increasing recognition of the importance of psychological interventions for weight reduction in adults with overweight and obesity (22). Psychological interventions address the underlying psychological and behavioural factors that contribute to excessive weight (23). These interventions support individuals to develop skills and strategies to cope with stress, negative emotions, and cognitions, modify unhelpful behaviours, and promote healthy lifestyle changes (22, 23). They also aim to increase self-efficacy, self-regulation, and self-awareness, which are critical for long-term weight reduction and maintenance (24). By targeting the psychological and behavioural factors that contribute to overweight and obesity, these interventions may be more effective for long-term weight loss maintenance compared to diet and physical activity interventions alone (25). These

For peer review only - http://bmjopen.bmj.com/site/about/guidelines.xhtml

interventions may be delivered individually or in groups, in clinical, community, workplace, and online settings, and include a variety of components such as cognitive behaviour change, mindfulness, relaxation and motivation (23, 24).

As research in the field of weight reduction and management continues to evolve, it is important to identify novel interventions (26). This is particularly important for psychological interventions, which are often overlooked in favour of more traditional approaches such as diet and exercise (24). Several systematic reviews have been conducted to assess the effectiveness of psychological interventions for weight loss in overweight or obese adults (23, 24). However, these reviews are limited by population (including only adults with specific medical conditions), inclusion of interventions (including only one type of psychological therapy intervention), or being substantially outdated (23, 24, 27). With the rapid development of new technology (e.g., digital health technologies) and therapeutic techniques, coupled with the ever-increasing rates of overweight and obesity, a scoping review is now needed to map the different types of psychological interventions and their core characteristics, and identify evidence gaps and areas for future research in this field (28).

Aim

To explore psychological interventions for adults with overweight or obesity as a means of achieving sustained weight reduction.

Objectives

1. To generate a comprehensive map of the types of psychological interventions for weight reduction and sustained weight reduction in adults with overweight or obesity.
2. To identify the core intervention characteristics including theoretical foundations, techniques, setting, duration, mode, number of sessions and provider of psychological interventions for weight reduction and sustained weight reduction in adults with overweight or obesity.
3. To identify study characteristics such as length of follow up, and outcomes of studies which include psychological interventions for weight reduction and sustained weight reduction in adults with overweight or obesity.
4. To generate a comprehensive map of the types of usual care for weight reduction and sustained weight reduction in adults with overweight or obesity.
5. To identify the core characteristics including components, setting, duration, mode, and providers of usual care for weight reduction and sustained weight reduction in adults with overweight or obesity.
6. To identify evidence gaps and areas for future research in the field of psychological interventions for weight reduction and sustained weight reduction in adults with overweight and obesity.

METHODS AND ANALYSIS

For peer review only - http://bmjopen.bmj.com/site/about/guidelines.xhtml

Study design

The study will follow a scoping review design, following the framework set out by Arksey and O'Malley, which recommends a five-stage review process (29):

- Stage 1. Identifying the research question.
- Stage 2. Identifying relevant studies.
- Stage 3. Study selection.
- Stage 4. Charting the data.
- Stage 5. Collating, summarising, and reporting the results

We will not implement the optional sixth stage set out by Arksey and O'Malley as the first five stages are adequate to satisfy the reviews aims and objectives (21). The study will be reported in accordance with the Preferred Reporting Items for Systematic reviews and Meta-Analyses extension for Scoping Reviews (PRISMA-ScR) guidelines (30).

A scoping review design was chosen because it enables an exploration of the broader spectrum of evidence relating to the range of psychological therapy interventions for adults with overweight and obesity, without being constrained by a rigid inclusion and exclusion criteria of a systematic review (28). Given the absence of previous reviews which have explored the objectives of this study, the scoping review methodology allows for a more expansive inclusion criteria (e.g., different study designs and intervention types) which will ensure that all relevant studies will be included. The scoping review design has been found to be well-suited for topics that are rapidly evolving and include a heterogeneous body of literature, which is applicable to psychological interventions as a means for achieving weight reduction and sustained weight reduction (in adults with overweight and obesity) (28).

Search strategy

Six databases will be searched to identify relevant articles:

1. Cochrane Central Register of Controlled Trials (CENTRAL).
2. World Health Organization International Clinical Trials Registry Platform (ICTRP).
3. EMBASE.
4. MEDLINE.
5. CINAHL.
6. PsycInfo.

We will search the above databases and trials registries from their inception to the present, with no limiters related to year of publication. A search of clinical trials will be conducted through the CENTRAL and ICTRP databases to identify ongoing clinical trials. We will check the reference lists of all included studies and relevant review articles for additional studies.

The search strategy conducted in each database will be based on the following:

- Population: Adults with overweight or obesity (aged ≥18, with BMI ≥25kg/m^2).

For peer review only - http://bmjopen.bmj.com/site/about/guidelines.xhtml

- Intervention: Psychological interventions (e.g., cognitive behaviour therapy, mindfulness, motivational interviewing etc).
- Study type: Randomised controlled trials (RCTs) or non-randomised comparative intervention studies.

The search strategy, designed by the research team in collaboration with an expert information specialist, will be adapted for each database (See Appendix A for Ovid example). The search strategy was based on the strategy outlined in the Cochrane review by Shaw et al (2005), but with the addition of further search terms identified by the research team, and the CADTH search filter (to identify randomised and non-randomised controlled trials) (24, 31).

Study selection

All articles identified by the electronic database searches will be imported into Rayyan (web application). One reviewer will then independently screen the titles and abstracts of the search results for appropriate studies. Following initial screening, two reviewers will independently screen the full texts of all potentially eligible studies. The two reviewers will resolve disagreements through discussion with a third reviewer. Full-text articles that meet the inclusion criteria will be charted to summarise the findings. The study selection process will be recorded in sufficient detail using a PRISMA flow diagram, reporting reasons for exclusion.

Data extraction

One reviewer will independently extract data for the included studies using a piloted data extraction form. A second reviewer will verify the data extraction. Data will be extracted on the following: study methods, participants, interventions (e.g., type, setting, components), outcomes and any other information judged to be important to meet the objectives of the study.

Inclusion criteria

While a scoping review traditionally encompasses a wide range of literature, specific criteria will be employed to identify relevant articles for the aim and objectives of the study.

Types of studies

Randomised controlled trials (RCTs) and non-randomised comparative intervention studies will be included. We will only include studies reported in full text. Whereby we identify an abstract meeting the inclusion criteria, we will contact the study authors to establish if a full text is available.

Types of participants

Studies will be included if the participants are ≥18 years and were classified as overweight or obese (BMI ≥ 25kg/m^2). Within studies, BMI, or the components of BMI, may be self-reported or measured. Studies will also be included when a sample includes participants with other classifications of BMI (e.g., underweight, healthy weight) if data relating to those with overweight or obesity can be extracted separately.

For peer review only - http://bmjopen.bmj.com/site/about/guidelines.xhtml

Studies will be included whereby participants with overweight or obesity have other co-morbidities (e.g., musculoskeletal pain, osteoarthritis, diabetes, hypertension, arthritis, hyperuricemia, gall bladder disease etc.), as BMI greater than 25kgm^2 has been associated with the incidence of multiple co-morbidities (32).

Types of interventions

Psychological therapy interventions will be defined as those interventions that involve meeting with a therapist (healthcare professional competent in providing psychological therapy) to discuss feelings and thoughts and how these affect behaviour and wellbeing (33). All psychological interventions will be considered for inclusion. Some of these interventions include cognitive behaviour therapy (CBT), mindfulness, psychodynamic therapies, motivational interviewing (MI), prolonged exposure therapy (PE), hypnotherapy, relaxation, psychotherapy, rationale emotional therapy and systematic desensitisation. Individual and group therapies will be included.

We will only include studies of psychological therapies that are designed to reduce weight or increase health promoting behaviour for the purpose of sustained weight reduction (i.e., eating behaviour, or physical activity).

To identify psychological interventions for weight reduction and sustained weight reduction, we will include studies with the following comparisons:

- Psychological therapy versus no intervention.
- Psychological therapy versus usual care (as defined by study authors).
- Comparisons between different types of psychological therapy (e.g., CBT, mindfulness, motivational interviewing etc).

We will not compare psychological interventions with other active interventions (e.g., diet and exercise interventions), unless these are classified as usual care.

Outcomes

Information on the following study outcomes, where available, will be collected: body weight or indicator of body mass (e.g., body mass index), adverse events, quality of life, physical activity levels and eating behaviours (e.g., constructs of dysfunctional, uncontrolled, restrained, emotional and adaptive eating behaviours as measured by instruments such as the Emotional Eating Scale, Intuitive Eating Scale-2, Mindfulness Eating Questionnaire, Dutch Eating Behaviour Questionnaire, Three-Factor Eating Questionnaire, etc (34-38).

Exclusion criteria

We will exclude studies that are not available in English, are not full text articles, and not peer reviewed articles (e.g., magazine articles, letters, editorials, newspaper, and commentary articles).

For peer review only - http://bmjopen.bmj.com/site/about/guidelines.xhtml

Data synthesis and analysis

A narrative synthesis will be employed to synthesise the data, prioritising and ordering data using the guidelines of the Synthesis WIthout Meta-analysis in systematic reviews (SWiM) (39).

Patient and public involvement (PPI)

A public advisor with more than ten years' experience of living with obesity has been involved in designing the review and this protocol paper, and will continue to have input throughout the project, including the determination of the objectives and improving the lay interpretability of the review. The public advisor will also help to co-develop the dissemination strategy.

What the findings may add

This scoping review is likely to identify specific gaps within existing evidence that may be useful in shaping future research initiatives. The identification of these gaps could pave the way for the development of novel innovative psychological interventions and may provide a roadmap for researchers to explore the effectiveness for these interventions. The findings of this review may also assist healthcare providers and key public health organisations gain insight into the types of psychological interventions that can be utilised as a means of weight reduction and sustained weight reduction for adults with overweight and obesity. With a greater understanding of psychological interventions (i.e., the types, characteristics, and impact) for weight reduction and sustaining weight reduction, key organisations and healthcare services can tailor programs to include evidenced based psychological components.

ETHICS AND DISSEMINATION

Ethical approval is not required to conduct this scoping review. The findings will be submitted to a peer-reviewed journal for publication. A lay summary will also be prepared with the assistance of the PPI author and disseminated on social media and to other key stakeholders involved in the care of adults with weight concerns. Findings will also be shared on the National Institute for Health and Care Research, Applied Research Collaboration - Northwest Coast (NIHR ARC-NWC) social media pages.

FUNDING STATEMENT

This work was partly funded by the National Institute for Health and Care Research Applied Research Collaboration North West Coast (NIHR ARC NWC). The work was also partly funded by the University of Central Lancashire, Research Institute for Global Health and Wellbeing (LIFE). The views expressed are those of the authors and not necessarily those of the NHS, the NIHR, or the Department of Health and Social Care.

CONFLICT OF INTERESTS STATEMENT

The authors have no conflicts of interest to declare.

For peer review only - http://bmjopen.bmj.com/site/about/guidelines.xhtml

AUTHOR STATEMENT - CONTRIBUTIONS

OH was responsible for the conception, design and writing of the manuscript. CW, EB, and AC provided support throughout the conception and design. AC, EB and JK contributed to the writing of the manuscript. AB provided PPI input throughout the development of the protocol, including the outcomes and background. CH designed the search strategy and performed the preliminary database searches. ES provided clinical input to support the development of the protocol. All authors read and approved the final manuscript.

REFERENCES

1. Cruickshank R, Crummey A, Muir VH. Obesity: a growing problem. Br Dent J. 2021;230(11):687-8.
2. Jastreboff AM, Kotz CM, Kahan S, Kelly AS, Heymsfield SB. Obesity as a disease: the obesity society 2018 position statement. Obesity. 2019;27(1):7-9.
3. Allison DB, Downey M, Atkinson RL, Billington CJ, Bray GA, Eckel RH, et al. Obesity as a disease: a white paper on evidence and arguments commissioned by the Council of the Obesity Society. Obesity. 2008;16(6):1161.
4. Burki T. European Commission classifies obesity as a chronic disease. The Lancet Diabetes & Endocrinology. 2021;9(7):418.
5. Kushner R. Obesity 2021: Current Clinical Management of a Chronic, Serious Disease. J Fam Pract. 2021;70(6s):S35-s40.
6. Sørensen TIA, Martinez AR, Jørgensen TSH. Epidemiology of Obesity. Handb Exp Pharmacol. 2022;274:3-27.
7. Dowd JB, Zajacova A. Long-term obesity and cardiovascular, inflammatory, and metabolic risk in U.S. adults. Am J Prev Med. 2014;46(6):578-84.
8. Bhaskaran K, Douglas I, Forbes H, dos-Santos-Silva I, Leon DA, Smeeth L. Body-mass index and risk of 22 specific cancers: a population-based cohort study of 5·24 million UK adults. Lancet. 2014;384(9945):755-65.
9. Martin-Rodriguez E, Guillen-Grima F, Martí A, Brugos-Larumbe A. Comorbidity associated with obesity in a large population: The APNA study. Obes Res Clin Pract. 2015;9(5):435-47.
10. Hill JO, Wyatt HR, Peters JC. Energy balance and obesity. Circulation. 2012;126(1):126-32.
11. de Lannoy L, Cowan T, Fernandez A, Ross R. Physical activity, diet, and weight loss in patients recruited from primary care settings: An update on obesity management interventions. Obes Sci Pract. 2021;7(5):619-28.
12. Hall KD, Kahan S. Maintenance of Lost Weight and Long-Term Management of Obesity. Med Clin North Am. 2018;102(1):183-97.
13. Chooi YC, Ding C, Magkos F. The epidemiology of obesity. Metabolism. 2019;92:6-10.
14. Deslippe AL, Soanes A, Bouchaud CC, Beckenstein H, Slim M, Plourde H, et al. Barriers and facilitators to diet, physical activity and lifestyle behavior intervention adherence: a qualitative systematic review of the literature. International Journal of Behavioral Nutrition and Physical Activity. 2023;20(1):14.
15. Kumar R, Rizvi MR, Saraswat S. Obesity and Stress: A Contingent Paralysis. Int J Prev Med. 2022;13:95.
16. Puhl RM, Heuer CA. Obesity stigma: important considerations for public health. Am J Public Health. 2010;100(6):1019-28.
17. Hamer O, Larkin, D., Relph, N., & Dey, P. . Fear as a barrier to physical activity in young adults with obesity: a qualitative study. . Qualitative Research in Sport, Exercise and Health. 2021.
18. McEwen B, Sapolsky R. Stress and Your Health. The Journal of Clinical Endocrinology & Metabolism. 2006;91(2):E2-E.
19. Torres SJ, Nowson CA. Relationship between stress, eating behavior, and obesity. Nutrition. 2007;23(11-12):887-94.

For peer review only - http://bmjopen.bmj.com/site/about/guidelines.xhtml

20. Hruby A, Hu FB. The Epidemiology of Obesity: A Big Picture. Pharmacoeconomics. 2015;33(7):673-89.
21. Hamer O, Larkin D, Relph N, Dey P. Fear-related barriers to physical activity among adults with overweight and obesity: A narrative synthesis scoping review. Obes Rev. 2021;22(11):e13307.
22. Barrett S, Begg S, O'Halloran P, Kingsley M. Integrated motivational interviewing and cognitive behaviour therapy for lifestyle mediators of overweight and obesity in community-dwelling adults: a systematic review and meta-analyses. BMC Public Health. 2018;18(1):1160.
23. Jacob A, Moullec G, Lavoie KL, Laurin C, Cowan T, Tisshaw C, et al. Impact of cognitive-behavioral interventions on weight loss and psychological outcomes: A meta-analysis. Health Psychol. 2018;37(5):417-32.
24. Shaw K, O'Rourke P, Del Mar C, Kenardy J. Psychological interventions for overweight or obesity. Cochrane Database Syst Rev. 2005(2):Cd003818.
25. Teixeira PJ, Carraça EV, Marques MM, Rutter H, Oppert J-M, De Bourdeaudhuij I, et al. Successful behavior change in obesity interventions in adults: a systematic review of self-regulation mediators. BMC Medicine. 2015;13(1):84.
26. Gómez Puente JM, Martínez-Marcos M. Overweight and obesity: effectiveness of interventions in adults. Enferm Clin (Engl Ed). 2018;28(1):65-74.
27. Palavras MA, Hay P, Filho CA, Claudino A. The Efficacy of Psychological Therapies in Reducing Weight and Binge Eating in People with Bulimia Nervosa and Binge Eating Disorder Who Are Overweight or Obese-A Critical Synthesis and Meta-Analyses. Nutrients. 2017;9(3).
28. Munn Z, Peters MDJ, Stern C, Tufanaru C, McArthur A, Aromataris E. Systematic review or scoping review? Guidance for authors when choosing between a systematic or scoping review approach. BMC Med Res Methodol. 2018;18(1):143.
29. Arksey H, O'Malley L. Scoping studies: towards a methodological framework. International Journal of Social Research Methodology. 2005;8:19 - 32.
30. Peters MDJ, Marnie C, Tricco AC, Pollock D, Munn Z, Alexander L, et al. Updated methodological guidance for the conduct of scoping reviews. JBI Evid Synth. 2020;18(10):2119-26.
31. CADTH. CADTH Search Filters Database CADTH Evidence Driven2023 [Available from: https://searchfilters.cadth.ca/link/34. .
32. Pi-Sunyer FX. Comorbidities of overweight and obesity: current evidence and research issues. Med Sci Sports Exerc. 1999;31(11 Suppl):S602-8.
33. NICE. Information for the public - Psychological therapy Online: National Institute for Health and Care Excellence; 2014 [Available from: https://www.nice.org.uk/guidance/cg178/ifp/chapter/psychological-therapy.
34. Van Strien T, Frijters JE, Bergers GP, Defares PB. The Dutch Eating Behavior Questionnaire (DEBQ) for assessment of restrained, emotional, and external eating behavior. International journal of eating disorders. 1986;5(2):295-315.
35. Cappelleri JC, Bushmakin AG, Gerber RA, Leidy NK, Sexton CC, Lowe MR, et al. Psychometric analysis of the Three-Factor Eating Questionnaire-R21: results from a large diverse sample of obese and non-obese participants. International Journal of Obesity. 2009;33(6):611-20.
36. Arnow B, Kenardy J, Agras WS. The emotional eating scale: The development of a measure to assess coping with negative affect by eating. International Journal of Eating Disorders. 1995;18(1):79-90.
37. Tylka TL, Kroon Van Diest AM. The Intuitive Eating Scale-2: item refinement and psychometric evaluation with college women and men. J Couns Psychol. 2013;60(1):137-53.
38. Framson C, Kristal AR, Schenk JM, Littman AJ, Zeliadt S, Benitez D. Development and Validation of the Mindful Eating Questionnaire. Journal of the American Dietetic Association. 2009;109(8):1439-44.
39. Campbell M, McKenzie JE, Sowden A, Katikireddi SV, Brennan SE, Ellis S, et al. Synthesis without meta-analysis (SWiM) in systematic reviews: reporting guideline. BMJ. 2020;368:l6890.

For peer review only

For peer review only

For peer review only - http://bmjopen.bmj.com/site/about/guidelines.xhtml

Supplementary material 1

APPENDIX A

Example search strategy

<u>Ovid MEDLINE(R) ALL <1946 to February 27, 2023></u>

1 Obesity/ 211963

2 Adiposity/ 15199

3 Weight Gain/ 35111

4 Weight Loss/ 42881

5 body mass index/ 147269

6 (obes* or adipos* or overweight* or over weight*).tw. 466238

7 (weight adj (reduc* or loss or losing or lose or maint* or decreas* or watch* or diet* or control* or manage*)).tw. 127938

8 or/1-7 672434

9 exp Psychotherapy/ 216317

10 (psychological therap* or psychological service* or psychological intervention* or psychological treatment* or psychotherap*).tw. 61353

11 counse?ling.tw. 112154

12 (interpersonal adj therap*).tw. 434

13 aversion therap*.tw. 187

14 behavio?r therap*.tw. 8063

15 behavio?r modific*.tw. 3108

16 colo?r therap*.tw. 27

17 (cognitiv* adj1 therap*).tw. 4261

18 gestalt therap*.tw. 94

19 music therap*.tw. 3010

20 milieu therap*.tw. 314

21 (assert* adj training).tw. 324

22 (nondirectiv* therap* or non directiv* therap*).tw. 47

23 ((problem solving or problemsolving) adj therap*).tw. 504

24 ((self control or selfcontrol) adj therap*).tw. 22

25 person cent*.tw. 8676

26 client cent*.tw. 1829

27 paradoxic* techni*.tw. 7

28 rational emoti*.tw. 289

29 reality therap*.tw. 189

30 (relax* adj train*).tw. 1436

31 mindfulness.tw. 11581

32 (sociotherap* or socio therap*).tw. 288

33 (socioenvironment* or socio environment*).tw. 1681

34 supportiv* therap*.tw. 5428

35 humanistic therap*.tw. 26

36 (person centered therap* or person centred therap*).tw. 42

37 psychodynamic therap*.tw. 619

38 motivational interviewing.tw. 4826

39 hypnotherapy.tw. 1216

40 or/9-39376330

41 (Randomized Controlled Trial or Controlled Clinical Trial or Pragmatic Clinical Trial or Clinical Study or Adaptive Clinical Trial or Equivalence Trial).pt. 684949

For peer review only

42 (Clinical Trial or Clinical Trial, Phase I or Clinical Trial, Phase II or Clinical Trial, Phase III or Clinical Trial, Phase IV or Clinical Trial Protocol).pt. 608099
43 Multicenter Study.pt. 331074
44 Clinical Studies as Topic/ 782
45 exp Clinical Trial/ or exp Clinical Trials as Topic/ or Clinical Trial Protocol/ or Clinical Trial Protocols as Topic/ or exp "Clinical Trial (topic)"/ 1265276
46 Multicenter Study/ or Multicenter Studies as Topic/ or "Multicenter Study (topic)"/ 350118
47 Randomization/ 106905
48 Random Allocation/ 106905
49 Double-Blind Method/ 174454
50 Double Blind Procedure/ 0
51 Double-Blind Studies/174454
52 Single-Blind Method/ 32528
53 Single Blind Procedure/ 0
54 Single-Blind Studies/ 32528
55 Placebos/ 35925
56 Placebo/ 0
57 Control Groups/ 1911
58 Control Group/ 1911
59 Cross-Over Studies/ or Crossover Procedure/ 54748
60 (random* or sham or placebo*).ti,ab,hw,kf. 1779092
61 ((singl* or doubl*) adj (blind* or dumm* or mask*)).ti,ab,hw,kf. 264573
62 ((tripl* or trebl*) adj (blind* or dumm* or mask*)).ti,ab,hw,kf. 1544
63 (control* adj3 (study or studies or trial* or group*)).ti,ab,hw,kf. 1894433
64 (clinical adj3 (study or studies or trial*)).ti,ab,hw,kf. 1411390
65 (Nonrandom* or non random* or non-random* or quasi-random* or quasirandom*).ti,ab,hw,kf. 53387
66 (phase adj3 (study or studies or trial*)).ti,ab,hw,kf. 174884
67 ((crossover or cross-over) adj3 (study or studies or trial*)).ti,ab,hw,kf. 75902
68 ((multicent* or multi-cent*) adj3 (study or studies or trial*)).ti,ab,hw,kf. 401960
69 allocated.ti,ab,hw. 81914
70 ((open label or open-label) adj5 (study or studies or trial*)).ti,ab,hw,kf. 43646
71 ((equivalence or superiority or non-inferiority or noninferiority) adj3 (study or studies or trial*)).ti,ab,hw,kf. 11683
72 (pragmatic study or pragmatic studies).ti,ab,hw,kf. 575
73 ((pragmatic or practical) adj3 trial*).ti,ab,hw,kf. 7471
74 ((quasiexperimental or quasi-experimental) adj3 (study or studies or trial*)).ti,ab,hw,kf. 11671
75 trial.ti,kf. 300216
76 or/41-75 3732550
77 8 and 40 and 76 5462
78 limit 77 to English language 5302

For peer review only

Psychological interventions for weight reduction and sustained weight reduction in adults with overweight and obesity: a scoping review protocol.

Journal:	*BMJ Open*
Manuscript ID	bmjopen-2023-075364.R2
Article Type:	Protocol
Date Submitted by the Author:	11-Oct-2023
Complete List of Authors:	Hamer, Oliver; University of Central Lancashire<br>Bray, Emma; University of Central Lancashire, Primary Care Clinical Sciences<br>Harris, Cath; University of Central Lancashire,<br>Blundell, Amy; University of Central Lancashire<br>Kuroski, Jennifer A; University of Central Lancashire<br>Schneider, Emma; Liverpool University Hospitals NHS Foundation Trust<br>Watkins, Caroline; University of Central Lancashire, Faculty of Health and Wellbeing<br>Clegg, Andrew; University of Central Lancashire
**Primary Subject Heading**:	Public health
Secondary Subject Heading:	Public health, Global health, Epidemiology
Keywords:	Obesity, PUBLIC HEALTH, Quality of Life, PSYCHIATRY, Systematic Review, Clinical Decision-Making

SCHOLARONE™
Manuscripts

Title page – BMJ Open

TITLE

Psychological interventions for weight reduction and sustained weight reduction in adults with overweight and obesity: a scoping review protocol.

AUTHORS

1. *Dr Oliver Hamer, Senior RA, University of Central Lancashire, Preston, Lancashire, PR1 2HE. United Kingdom.
2. Dr Emma Bray, Senior Research Fellow, University of Central Lancashire, Preston, Lancashire, PR1 2HE. United Kingdom.
3. Catherine Harris, Information Specialist, University of Central Lancashire, Preston, Lancashire, PR1 2HE. United Kingdom.
4. Amy Blundell, Public Advisor, University of Central Lancashire - Comensus patient and public advisor group, Preston, Lancashire, PR1 2HE. United Kingdom.
5. Jennifer Kuroski, Research Associate, University of Central Lancashire, Preston, Lancashire, PR1 2HE. United Kingdom.
6. Emma Schneider, Advanced weight Management Specialist, Liverpool University Hospitals NHS Foundation Trust, Prescot Street, Liverpool, Merseyside, L7 8XP
7. Professor Dame Caroline Watkins, Professor of Stroke and Older People's Care, University of Central Lancashire, Preston, Lancashire, PR1 2HE. United Kingdom.
8. Professor Andrew Clegg, Professor of Health Services Research, University of Central Lancashire, Preston, Lancashire, PR1 2HE. United Kingdom.

*Corresponding author, Oliver Hamer, OHamer@uclan.ac.uk

KEYWORDS

Obesity; Body weight; Weight loss; Psychological intervention; Scoping review.

ABSTRACT

Introduction: Overweight and obesity are growing public health problems worldwide. Both diet and physical activity have been the primary interventions for weight reduction over the past decade. With increasing rates of overweight and obesity, it is evident that a primary focus on diet and exercise has not resulted in sustained obesity reduction within the global population. There is now a case to explore other weight management strategies, focusing on psychological factors that may underpin overweight and obesity. Psychological therapy interventions are gaining recognition for their effectiveness in addressing underlying emotional factors and promoting weight loss. However, there is a dearth of literature that has mapped the types of psychological interventions and the characteristics of these interventions as a means of achieving weight reduction and sustained weight reduction in adults with overweight or obesity.

Methods and analysis: The review will combine the methodology outlined by Arksey and O'Malley with the PRISMA-ScR guidelines. A total of six databases will be searched using a comprehensive search strategy. Intervention studies will be included if participants are 18 years and over, classified as overweight or obese (≥BMI 25kg/m^2), and have received a

For peer review only - http://bmjopen.bmj.com/site/about/guidelines.xhtml

psychological therapy intervention. The review will exclude studies that are not available in English, not full text, none peer reviewed or combine a lifestyle and/or pharmacological intervention with a psychological intervention. Data will be synthesised using a narrative synthesis approach.

Ethics and dissemination: Ethical approval is not required to conduct this scoping review. The findings will be disseminated through journal publication(s), social media, and a lay summary for key stakeholders.

Strengths and limitations of this study

- A comprehensive literature search of six electronic databases has been designed collaboratively with a skilled information specialist.
- The protocol for the review has been co-developed with a public advisor who has over ten years' experience living with obesity.
- The scoping review will be limited to comparisons with other psychological interventions, usual care, or no intervention, and will not therefore include a comparison with other active lifestyle interventions (unless classified as usual care).
- Relevant papers written in other languages could be omitted from this review, given the inclusion criteria to only include papers written in English.

WORDCOUNT

2498

For peer review only

Manuscript – BMJ Open

INTRODUCTION

Obesity is a growing public health problem worldwide (1). It has recently been recognised (and classified) by The Obesity Society and the European Commission as a chronic disease (2-5). Globally, approximately 650 million adults and 340 million children and adolescents (ages 5-19) currently suffer from obesity (6). Obesity is known to increase the risks of a wide spectrum of comorbidities, including cardiovascular disease, type 2 diabetes, cancer, hypertension, and several types of musculoskeletal disorders (7-9). It has a multifaceted aetiology, with factors such as genetics, medical conditions, stress and environmental factors contributing to the prevalence of obesity (1).

Energy balance (energy input from food consumption and energy expenditure from physical activity) is one of the key factors to weight maintenance and, hence, diet (energy intake) and physical activity levels (energy output) are key behaviour determinants of obesity (10). Over the last 20 years, diet and physical activity have been the primary interventions recommended for weight loss (11). However, despite the efficacy of these interventions, long-term weight loss maintenance remains a challenge, with many individuals regaining lost weight. (12) With worldwide prevalence of overweight and obesity rates continuing to rise, it is evident that dietary and physical activity interventions have not resulted in a sustained obesity reduction within the global population (13). The lack of longer term (or population) success associated with these interventions may be due to a primary focus on increasing energy expenditure and reducing dietary intake (14). While these factors are critical for weight loss, they ignore the complex psychological factors (e.g., emotional, such as fears and behavioural, such as avoidance) that underpin overweight and obesity (6, 15-18). These factors can contribute to disordered eating, low self-esteem, depression, anxiety, and stress (which may lead to weight gain) (15, 19). Furthermore, these interventions often ignore the environmental factors that contribute to weight gain, such as food accessibility, social support, and cultural norms (20). With the previous emphasis on diet and physical activity interventions seemingly having little impact on population prevalence of overweight and obesity, there is now a case to explore additional weight management strategies, focusing on the psychological factors that may underpin overweight and obesity (17, 21).

In recent years, there has been increasing recognition of the importance of psychological interventions for weight reduction in adults with overweight and obesity (22). Psychological interventions address the underlying psychological and behavioural factors that contribute to excessive weight (23). These interventions support individuals to develop skills and strategies to cope with stress, negative emotions, and cognitions, modify unhelpful behaviours, and promote healthy lifestyle changes (22, 23). They also aim to increase self-efficacy, self-regulation, and self-awareness, which are critical for long-term weight reduction and maintenance (24). By targeting the psychological and behavioural factors that contribute to overweight and obesity, these interventions may be more effective for long-term weight loss maintenance compared to diet and physical activity interventions alone (25). These

For peer review only - http://bmjopen.bmj.com/site/about/guidelines.xhtml

interventions may be delivered individually or in groups, in clinical, community, workplace, and online settings, and include a variety of components such as cognitive behaviour change, mindfulness, relaxation and motivation (23, 24).

As research in the field of weight reduction and management continues to evolve, it is important to identify novel interventions (26). This is particularly important for psychological interventions, which are often overlooked in favour of more traditional approaches such as diet and exercise (24). Several systematic reviews have been conducted to assess the effectiveness of psychological interventions for weight loss in overweight or obese adults (23, 24). However, these reviews are limited by population (including only adults with specific medical conditions), inclusion of interventions (including only one type of psychological therapy intervention), or being substantially outdated (23, 24, 27). With the rapid development of new technology (e.g., digital health technologies) and therapeutic techniques, coupled with the ever-increasing rates of overweight and obesity, a scoping review is now needed to map the different types of psychological interventions and their core characteristics, and identify evidence gaps and areas for future research in this field (28).

Aim

To explore psychological interventions for adults with overweight or obesity as a means of achieving sustained weight reduction.

Objectives

1. To generate a comprehensive map of the types of psychological interventions for weight reduction and sustained weight reduction in adults with overweight or obesity.
2. To identify the core intervention characteristics including theoretical foundations, techniques, setting, duration, mode, number of sessions and provider of psychological interventions for weight reduction and sustained weight reduction in adults with overweight or obesity.
3. To identify study characteristics such as length of follow up, and outcomes of studies which include psychological interventions for weight reduction and sustained weight reduction in adults with overweight or obesity.
4. To generate a comprehensive map of the types of usual care for weight reduction and sustained weight reduction in adults with overweight or obesity.
5. To identify the core characteristics including components, setting, duration, mode, and providers of usual care for weight reduction and sustained weight reduction in adults with overweight or obesity.
6. To identify evidence gaps and areas for future research in the field of psychological interventions for weight reduction and sustained weight reduction in adults with overweight and obesity.

METHODS AND ANALYSIS

For peer review only - http://bmjopen.bmj.com/site/about/guidelines.xhtml

Study design

The study will follow a scoping review design, following the framework set out by Arksey and O'Malley, which recommends a five-stage review process (29):

- Stage 1. Identifying the research question.
- Stage 2. Identifying relevant studies.
- Stage 3. Study selection.
- Stage 4. Charting the data.
- Stage 5. Collating, summarising, and reporting the results

We will not implement the optional sixth stage set out by Arksey and O'Malley as the first five stages are adequate to satisfy the reviews aims and objectives (21). The study will be reported in accordance with the Preferred Reporting Items for Systematic reviews and Meta-Analyses extension for Scoping Reviews (PRISMA-ScR) guidelines (30).

A scoping review design was chosen because it enables an exploration of the broader spectrum of evidence relating to the range of psychological therapy interventions for adults with overweight and obesity, without being constrained by a rigid inclusion and exclusion criteria of a systematic review (28). Given the absence of previous reviews which have explored the objectives of this study, the scoping review methodology allows for a more expansive inclusion criteria (e.g., different study designs and intervention types) which will ensure that all relevant studies will be included. The scoping review design has been found to be well-suited for topics that are rapidly evolving and include a heterogeneous body of literature, which is applicable to psychological interventions as a means for achieving weight reduction and sustained weight reduction (in adults with overweight and obesity) (28).

Search strategy

Six databases will be searched to identify relevant articles:

1. Cochrane Central Register of Controlled Trials (CENTRAL).
2. World Health Organization International Clinical Trials Registry Platform (ICTRP).
3. EMBASE.
4. MEDLINE.
5. CINAHL.
6. PsycInfo.

We will search the above databases and trials registries from their inception to the present, with no limiters related to year of publication. A search of clinical trials will be conducted through the CENTRAL and ICTRP databases to identify ongoing clinical trials. We will check the reference lists of all included studies and relevant review articles for additional studies.

The search strategy conducted in each database will be based on the following:

- Population: Adults with overweight or obesity (aged ≥18, with BMI ≥25kg/m^2).

For peer review only - http://bmjopen.bmj.com/site/about/guidelines.xhtml

- Intervention: Psychological interventions (e.g., cognitive behaviour therapy, mindfulness, motivational interviewing etc).
- Study type: Randomised controlled trials (RCTs) or non-randomised comparative intervention studies.

The search strategy, designed by the research team in collaboration with an expert information specialist, will be adapted for each database (See Appendix A for Ovid example). The search strategy was based on the strategy outlined in the Cochrane review by Shaw et al (2005), but with the addition of further search terms identified by the research team, and the CADTH search filter (to identify randomised and non-randomised controlled trials) (24, 31).

Study selection

All articles identified by the electronic database searches will be imported into Rayyan (web application). One reviewer will then independently screen the titles and abstracts of the search results for appropriate studies. Following initial screening, two reviewers will independently screen the full texts of all potentially eligible studies. The two reviewers will resolve disagreements through discussion with a third reviewer. Full-text articles that meet the inclusion criteria will be charted to summarise the findings. The study selection process will be recorded in sufficient detail using a PRISMA flow diagram, reporting reasons for exclusion.

Data extraction

One reviewer will independently extract data for the included studies using a piloted data extraction form. A second reviewer will verify the data extraction. Data will be extracted on the following: study methods, participants, interventions (e.g., type, setting, components), outcomes and any other information judged to be important to meet the objectives of the study.

Inclusion criteria

While a scoping review traditionally encompasses a wide range of literature, specific criteria will be employed to identify relevant articles for the aim and objectives of the study.

Types of studies

Randomised controlled trials (RCTs) and non-randomised comparative intervention studies will be included. We will only include studies reported in full text. Whereby we identify an abstract meeting the inclusion criteria, we will contact the study authors to establish if a full text is available.

Types of participants

Studies will be included if the participants are ≥18 years and were classified as overweight or obese (BMI ≥ 25kg/m^2). Within studies, BMI, or the components of BMI, may be self-reported or measured. Studies will also be included when a sample includes participants with other classifications of BMI (e.g., underweight, healthy weight) if data relating to those with overweight or obesity can be extracted separately.

For peer review only - http://bmjopen.bmj.com/site/about/guidelines.xhtml

For peer review only

Studies will be included whereby participants with overweight or obesity have other co-morbidities (e.g., musculoskeletal pain, osteoarthritis, diabetes, hypertension, arthritis, hyperuricemia, gall bladder disease etc.), as BMI greater than 25kgm^2 has been associated with the incidence of multiple co-morbidities (32).

Types of interventions

Psychological therapy interventions will be defined as those interventions that involve meeting with a therapist (healthcare professional competent in providing psychological therapy) to discuss feelings and thoughts and how these affect behaviour and wellbeing (33). All psychological interventions will be considered for inclusion. Some of these interventions include cognitive behaviour therapy (CBT), mindfulness, psychodynamic therapies, motivational interviewing (MI), prolonged exposure therapy (PE), hypnotherapy, relaxation, psychotherapy, rationale emotional therapy and systematic desensitisation. Individual and group therapies will be included.

We will only include studies of psychological therapies that are designed to reduce weight or increase health promoting behaviour for the purpose of sustained weight reduction (i.e., eating behaviour, or physical activity).

To identify psychological interventions for weight reduction and sustained weight reduction, we will include studies with the following comparisons:

- Psychological therapy versus no intervention.
- Psychological therapy versus usual care (as defined by study authors).
- Comparisons between different types of psychological therapy (e.g., CBT, mindfulness, motivational interviewing etc).

We will not compare psychological interventions with other active interventions (e.g., diet and exercise interventions), unless these are classified as usual care.

Outcomes

Information on the following study outcomes, where available, will be collected: body weight or indicator of body mass (e.g., body mass index), adverse events, quality of life, physical activity levels and eating behaviours (e.g., constructs of dysfunctional, uncontrolled, restrained, emotional and adaptive eating behaviours as measured by instruments such as the Emotional Eating Scale, Intuitive Eating Scale-2, Mindfulness Eating Questionnaire, Dutch Eating Behaviour Questionnaire, Three-Factor Eating Questionnaire, etc (34-38).

Exclusion criteria

We will exclude studies that are not available in English, are not full text articles, and not peer reviewed articles (e.g., magazine articles, letters, editorials, newspaper, and commentary articles).

For peer review only - http://bmjopen.bmj.com/site/about/guidelines.xhtml

Data synthesis and analysis

A narrative synthesis will be employed to synthesise the data, prioritising and ordering data using the guidelines of the Synthesis WIthout Meta-analysis in systematic reviews (SWiM) (39).

Patient and public involvement (PPI)

A public advisor with more than ten years' experience of living with obesity has been involved in designing the review and this protocol paper, and will continue to have input throughout the project, including the determination of the objectives and improving the lay interpretability of the review. The public advisor will also help to co-develop the dissemination strategy.

What the findings may add

This scoping review is likely to identify specific gaps within existing evidence that may be useful in shaping future research initiatives. The identification of these gaps could pave the way for the development of novel innovative psychological interventions and may provide a roadmap for researchers to explore the effectiveness for these interventions. The findings of this review may also assist healthcare providers and key public health organisations gain insight into the types of psychological interventions that can be utilised as a means of weight reduction and sustained weight reduction for adults with overweight and obesity. With a greater understanding of psychological interventions (i.e., the types, characteristics, and impact) for weight reduction and sustaining weight reduction, key organisations and healthcare services can tailor programs to include evidenced based psychological components.

ETHICS AND DISSEMINATION

Ethical approval is not required to conduct this scoping review. The findings will be submitted to a peer-reviewed journal for publication. A lay summary will also be prepared with the assistance of the PPI author and disseminated on social media and to other key stakeholders involved in the care of adults with weight concerns. Findings will also be shared on the National Institute for Health and Care Research, Applied Research Collaboration - Northwest Coast (NIHR ARC-NWC) social media pages.

FUNDING STATEMENT

This work was partly funded by the National Institute for Health and Care Research Applied Research Collaboration North West Coast (NIHR ARC NWC). The work was also partly funded by the University of Central Lancashire, Research Institute for Global Health and Wellbeing (grant number: LIFE RSAC3-02). The views expressed are those of the authors and not necessarily those of the NHS, the NIHR, or the Department of Health and Social Care.

CONFLICT OF INTERESTS STATEMENT

For peer review only

The authors have no conflicts of interest to declare.

AUTHOR STATEMENT - CONTRIBUTIONS

OH was responsible for the conception, design and writing of the manuscript. CW, EB, and AC provided support throughout the conception and design. AC, EB and JK contributed to the writing of the manuscript. AB provided PPI input throughout the development of the protocol, including the outcomes and background. CH designed the search strategy and performed the preliminary database searches. ES provided clinical input to support the development of the protocol. All authors read and approved the final manuscript.

REFERENCES

1. Cruickshank R, Crummey A, Muir VH. Obesity: a growing problem. Br Dent J. 2021;230(11):687-8.
2. Jastreboff AM, Kotz CM, Kahan S, Kelly AS, Heymsfield SB. Obesity as a disease: the obesity society 2018 position statement. Obesity. 2019;27(1):7-9.
3. Allison DB, Downey M, Atkinson RL, Billington CJ, Bray GA, Eckel RH, et al. Obesity as a disease: a white paper on evidence and arguments commissioned by the Council of the Obesity Society. Obesity. 2008;16(6):1161.
4. Burki T. European Commission classifies obesity as a chronic disease. The Lancet Diabetes & Endocrinology. 2021;9(7):418.
5. Kushner R. Obesity 2021: Current Clinical Management of a Chronic, Serious Disease. J Fam Pract. 2021;70(6s):S35-s40.
6. Sørensen TIA, Martinez AR, Jørgensen TSH. Epidemiology of Obesity. Handb Exp Pharmacol. 2022;274:3-27.
7. Dowd JB, Zajacova A. Long-term obesity and cardiovascular, inflammatory, and metabolic risk in U.S. adults. Am J Prev Med. 2014;46(6):578-84.
8. Bhaskaran K, Douglas I, Forbes H, dos-Santos-Silva I, Leon DA, Smeeth L. Body-mass index and risk of 22 specific cancers: a population-based cohort study of 5·24 million UK adults. Lancet. 2014;384(9945):755-65.
9. Martin-Rodriguez E, Guillen-Grima F, Martí A, Brugos-Larumbe A. Comorbidity associated with obesity in a large population: The APNA study. Obes Res Clin Pract. 2015;9(5):435-47.
10. Hill JO, Wyatt HR, Peters JC. Energy balance and obesity. Circulation. 2012;126(1):126-32.
11. de Lannoy L, Cowan T, Fernandez A, Ross R. Physical activity, diet, and weight loss in patients recruited from primary care settings: An update on obesity management interventions. Obes Sci Pract. 2021;7(5):619-28.
12. Hall KD, Kahan S. Maintenance of Lost Weight and Long-Term Management of Obesity. Med Clin North Am. 2018;102(1):183-97.
13. Chooi YC, Ding C, Magkos F. The epidemiology of obesity. Metabolism. 2019;92:6-10.
14. Deslippe AL, Soanes A, Bouchaud CC, Beckenstein H, Slim M, Plourde H, et al. Barriers and facilitators to diet, physical activity and lifestyle behavior intervention adherence: a qualitative systematic review of the literature. International Journal of Behavioral Nutrition and Physical Activity. 2023;20(1):14.
15. Kumar R, Rizvi MR, Saraswat S. Obesity and Stress: A Contingent Paralysis. Int J Prev Med. 2022;13:95.
16. Puhl RM, Heuer CA. Obesity stigma: important considerations for public health. Am J Public Health. 2010;100(6):1019-28.
17. Hamer O, Larkin, D., Relph, N., & Dey, P. . Fear as a barrier to physical activity in young adults with obesity: a qualitative study. . Qualitative Research in Sport, Exercise and Health. 2021.
18. McEwen B, Sapolsky R. Stress and Your Health. The Journal of Clinical Endocrinology & Metabolism. 2006;91(2):E2-E.
19. Torres SJ, Nowson CA. Relationship between stress, eating behavior, and obesity. Nutrition. 2007;23(11-12):887-94.

For peer review only - http://bmjopen.bmj.com/site/about/guidelines.xhtml

20. Hruby A, Hu FB. The Epidemiology of Obesity: A Big Picture. Pharmacoeconomics. 2015;33(7):673-89.
21. Hamer O, Larkin D, Relph N, Dey P. Fear-related barriers to physical activity among adults with overweight and obesity: A narrative synthesis scoping review. Obes Rev. 2021;22(11):e13307.
22. Barrett S, Begg S, O'Halloran P, Kingsley M. Integrated motivational interviewing and cognitive behaviour therapy for lifestyle mediators of overweight and obesity in community-dwelling adults: a systematic review and meta-analyses. BMC Public Health. 2018;18(1):1160.
23. Jacob A, Moullec G, Lavoie KL, Laurin C, Cowan T, Tisshaw C, et al. Impact of cognitive-behavioral interventions on weight loss and psychological outcomes: A meta-analysis. Health Psychol. 2018;37(5):417-32.
24. Shaw K, O'Rourke P, Del Mar C, Kenardy J. Psychological interventions for overweight or obesity. Cochrane Database Syst Rev. 2005(2):Cd003818.
25. Teixeira PJ, Carraça EV, Marques MM, Rutter H, Oppert J-M, De Bourdeaudhuij I, et al. Successful behavior change in obesity interventions in adults: a systematic review of self-regulation mediators. BMC Medicine. 2015;13(1):84.
26. Gómez Puente JM, Martínez-Marcos M. Overweight and obesity: effectiveness of interventions in adults. Enferm Clin (Engl Ed). 2018;28(1):65-74.
27. Palavras MA, Hay P, Filho CA, Claudino A. The Efficacy of Psychological Therapies in Reducing Weight and Binge Eating in People with Bulimia Nervosa and Binge Eating Disorder Who Are Overweight or Obese-A Critical Synthesis and Meta-Analyses. Nutrients. 2017;9(3).
28. Munn Z, Peters MDJ, Stern C, Tufanaru C, McArthur A, Aromataris E. Systematic review or scoping review? Guidance for authors when choosing between a systematic or scoping review approach. BMC Med Res Methodol. 2018;18(1):143.
29. Arksey H, O'Malley L. Scoping studies: towards a methodological framework. International Journal of Social Research Methodology. 2005;8:19 - 32.
30. Peters MDJ, Marnie C, Tricco AC, Pollock D, Munn Z, Alexander L, et al. Updated methodological guidance for the conduct of scoping reviews. JBI Evid Synth. 2020;18(10):2119-26.
31. CADTH. CADTH Search Filters Database CADTH Evidence Driven2023 [Available from: https://searchfilters.cadth.ca/link/34. .
32. Pi-Sunyer FX. Comorbidities of overweight and obesity: current evidence and research issues. Med Sci Sports Exerc. 1999;31(11 Suppl):S602-8.
33. NICE. Information for the public - Psychological therapy Online: National Institute for Health and Care Excellence; 2014 [Available from: https://www.nice.org.uk/guidance/cg178/ifp/chapter/psychological-therapy.
34. Van Strien T, Frijters JE, Bergers GP, Defares PB. The Dutch Eating Behavior Questionnaire (DEBQ) for assessment of restrained, emotional, and external eating behavior. International journal of eating disorders. 1986;5(2):295-315.
35. Cappelleri JC, Bushmakin AG, Gerber RA, Leidy NK, Sexton CC, Lowe MR, et al. Psychometric analysis of the Three-Factor Eating Questionnaire-R21: results from a large diverse sample of obese and non-obese participants. International Journal of Obesity. 2009;33(6):611-20.
36. Arnow B, Kenardy J, Agras WS. The emotional eating scale: The development of a measure to assess coping with negative affect by eating. International Journal of Eating Disorders. 1995;18(1):79-90.
37. Tylka TL, Kroon Van Diest AM. The Intuitive Eating Scale-2: item refinement and psychometric evaluation with college women and men. J Couns Psychol. 2013;60(1):137-53.
38. Framson C, Kristal AR, Schenk JM, Littman AJ, Zeliadt S, Benitez D. Development and Validation of the Mindful Eating Questionnaire. Journal of the American Dietetic Association. 2009;109(8):1439-44.
39. Campbell M, McKenzie JE, Sowden A, Katikireddi SV, Brennan SE, Ellis S, et al. Synthesis without meta-analysis (SWiM) in systematic reviews: reporting guideline. BMJ. 2020;368:l6890.

For peer review only

For peer review only

Supplementary material 1
APPENDIX A
Example search strategy

Ovid MEDLINE(R) ALL <1946 to February 27, 2023>

1 Obesity/ 211963
2 Adiposity/ 15199
3 Weight Gain/ 35111
4 Weight Loss/ 42881
5 body mass index/ 147269
6 (obes* or adipos* or overweight* or over weight*).tw. 466238
7 (weight adj (reduc* or loss or losing or lose or maint* or decreas* or watch* or diet* or control* or manage*)).tw. 127938
8 or/1-7 672434
9 exp Psychotherapy/ 216317
10 (psychological therap* or psychological service* or psychological intervention* or psychological treatment* or psychotherap*).tw. 61353
11 counse?ling.tw. 112154
12 (interpersonal adj therap*).tw. 434
13 aversion therap*.tw. 187
14 behavio?r therap*.tw. 8063
15 behavio?r modific*.tw. 3108
16 colo?r therap*.tw. 27
17 (cognitiv* adj1 therap*).tw. 4261
18 gestalt therap*.tw. 94
19 music therap*.tw. 3010
20 milieu therap*.tw. 314
21 (assert* adj training).tw. 324
22 (nondirectiv* therap* or non directiv* therap*).tw. 47
23 ((problem solving or problemsolving) adj therap*).tw. 504
24 ((self control or selfcontrol) adj therap*).tw. 22
25 person cent*.tw. 8676
26 client cent*.tw. 1829
27 paradoxic* techni*.tw. 7
28 rational emoti*.tw. 289
29 reality therap*.tw. 189
30 (relax* adj train*).tw. 1436
31 mindfulness.tw. 11581
32 (sociotherap* or socio therap*).tw. 288
33 (socioenvironment* or socio environment*).tw. 1681
34 supportiv* therap*.tw. 5428
35 humanistic therap*.tw. 26
36 (person centered therap* or person centred therap*).tw. 42
37 psychodynamic therap*.tw. 619
38 motivational interviewing.tw. 4826
39 hypnotherapy.tw. 1216
40 or/9-39376330
41 (Randomized Controlled Trial or Controlled Clinical Trial or Pragmatic Clinical Trial or Clinical Study or Adaptive Clinical Trial or Equivalence Trial).pt. 684949

For peer review only

42 (Clinical Trial or Clinical Trial, Phase I or Clinical Trial, Phase II or Clinical Trial, Phase III or Clinical Trial, Phase IV or Clinical Trial Protocol).pt. 608099
43 Multicenter Study.pt. 331074
44 Clinical Studies as Topic/ 782
45 exp Clinical Trial/ or exp Clinical Trials as Topic/ or Clinical Trial Protocol/ or Clinical Trial Protocols as Topic/ or exp "Clinical Trial (topic)"/ 1265276
46 Multicenter Study/ or Multicenter Studies as Topic/ or "Multicenter Study (topic)"/ 350118
47 Randomization/ 106905
48 Random Allocation/ 106905
49 Double-Blind Method/ 174454
50 Double Blind Procedure/ 0
51 Double-Blind Studies/174454
52 Single-Blind Method/ 32528
53 Single Blind Procedure/ 0
54 Single-Blind Studies/ 32528
55 Placebos/ 35925
56 Placebo/ 0
57 Control Groups/ 1911
58 Control Group/ 1911
59 Cross-Over Studies/ or Crossover Procedure/ 54748
60 (random* or sham or placebo*).ti,ab,hw,kf. 1779092
61 ((singl* or doubl*) adj (blind* or dumm* or mask*)).ti,ab,hw,kf. 264573
62 ((tripl* or trebl*) adj (blind* or dumm* or mask*)).ti,ab,hw,kf. 1544
63 (control* adj3 (study or studies or trial* or group*)).ti,ab,hw,kf. 1894433
64 (clinical adj3 (study or studies or trial*)).ti,ab,hw,kf. 1411390
65 (Nonrandom* or non random* or non-random* or quasi-random* or quasirandom*).ti,ab,hw,kf. 53387
66 (phase adj3 (study or studies or trial*)).ti,ab,hw,kf. 174884
67 ((crossover or cross-over) adj3 (study or studies or trial*)).ti,ab,hw,kf. 75902
68 ((multicent* or multi-cent*) adj3 (study or studies or trial*)).ti,ab,hw,kf. 401960
69 allocated.ti,ab,hw. 81914
70 ((open label or open-label) adj5 (study or studies or trial*)).ti,ab,hw,kf. 43646
71 ((equivalence or superiority or non-inferiority or noninferiority) adj3 (study or studies or trial*)).ti,ab,hw,kf. 11683
72 (pragmatic study or pragmatic studies).ti,ab,hw,kf. 575
73 ((pragmatic or practical) adj3 trial*).ti,ab,hw,kf. 7471
74 ((quasiexperimental or quasi-experimental) adj3 (study or studies or trial*)).ti,ab,hw,kf. 11671
75 trial.ti,kf. 300216
76 or/41-75 3732550
77 8 and 40 and 76 5462
78 limit 77 to English language 5302

For peer review only
